# Conserved Arginines of Bovine Adenovirus-3 33K Protein Are Important for Transportin-3 Mediated Transport and Virus Replication

**DOI:** 10.1371/journal.pone.0101216

**Published:** 2014-07-14

**Authors:** Vikas Kulshreshtha, Lisanework E. Ayalew, Azharul Islam, Suresh K. Tikoo

**Affiliations:** 1 VIDO-InterVac, University of Saskatchewan, Saskatoon, Saskatchewan, Canada; 2 Veterinary Microbiology, University of Saskatchewan, Saskatoon, Saskatchewan, Canada; 3 Vaccinology & Immunotherapeutics Program, School of Public Health, University of Saskatchewan, Saskatoon, Saskatchewan, Canada; University of Nebraska – Lincoln, United States of America

## Abstract

The L6 region of bovine adenovirus (BAdV)-3 encodes a spliced protein designated 33K. The 33K specific sera detected five major proteins and three minor proteins in transfected or virus infected cells, which could arise by internal initiation of translation and alternative splicing. The 33K protein is predominantly localized to the nucleus of BAdV-3 infected cells. The 33K nuclear transport utilizes both classical importin-α/-β and importin-β dependent nuclear import pathways and preferentially binds to importin-α5 and transportin-3 receptors, respectively. Analysis of mutant 33K proteins demonstrated that amino acids 201–240 of the conserved C-terminus of 33K containing RS repeat are required for nuclear localization and, binding to both importin-α5 and transportin-3 receptors. Interestingly, the arginine residues of conserved RS repeat are required for binding to transportin-3 receptor but not to importin-α5 receptor. Moreover, mutation of arginines residues of RS repeat proved lethal for production of progeny virus. Our results suggest that arginines of RS repeat are required for efficient nuclear transport of 33K mediated by transportin-3, which appears to be essential for replication and production of infectious virion.

## Introduction

Nuclear transport of proteins usually takes place by binding of protein nuclear localization signal (NLS) to carrier proteins located in the cytoplasm [1]. Specific recognition of a NLS by the carrier protein provide selective sorting of proteins. Most transport is mediated by karyopherins belonging to importin-β family including transportins (1,2, SR1, SR2), importins (4,5,7) and importin-β [2]. The classical nuclear import is mediated by importin-α/-β pathway where protein NLS is recognized by an adapter protein importin-α, which interacts with actual transport receptor importin-β forming a heterodimer complex which is translocated through nuclear pore complex. However, some of the proteins do not require adapter proteins (importin-α) and directly bind the import receptor (importin-β, importin-7, transportin) for translocating through nuclear pore complex for transport to nucleus [1], [2]. Interestingly, some viral proteins use both classical importin-α/-β mediated and importin-β family members mediated nuclear import pathways [3], [4], [5], [6].

Bovine adenovirus-3, a member of *Mastadenovirus* genus is a non enveloped virion with a linear double stranded DNA genome of 34446 bp [7]. BAdV-3 gene expression is temporally regulated, which divides genome into early, intermediate and late phases. The late region encodes structural and non structural proteins [7]. The L6 region of late transcriptional unit of BAdV-3 encodes three non structural proteins 33K, 22K and 100K [7], [8]. The L4 region of late transcriptional unit of HAdV-5 encodes 100K, 33K and 22K proteins, which are involved in different steps of adenovirus replication [9], [10], [11], [12] including regulation of late gene expression, post transcriptional regulation of gene expression and viral DNA encapsidation [13], [14].

Earlier, we demonstrated that BAdV-3 33K protein a) is a product of spliced transcript, which shares N-terminus 138 amino acids with 22K, b) interact with BAdV-3 pV and 100K protein, c) may be involved in virus assembly, and, d) N-terminus 97 amino acids shared by 33K and 22K may be involved in DNA encapsidation [8], [15], [16]. Here, we report the identification and characterization of pathways mediating nuclear transport of 33K, and demonstrate that arginines of RS motifs located in the C-terminal conserved region interact with transportin-3 and appear essential for BAdV-3 replication.

## Materials and Methods

### Cell lines and virus

Madin-Darby bovine kidney (MDBK) cells, 293T cells and VIDO DT1 cells [17] were cultured in minimal essential medium (MEM) (Gibco-BRL) containing 10% fetal bovine serum (FBS). HeLa cells were cultured in Dulbecco's modified MEM containing 10% FBS. The wild-type BAdV-3 was cultivated in MDBK cells as described [18].

### Plasmid construction

Plasmid pGST-TRN-SR2 was a gift from Dr. Woan-Yuh Tarn and has been previously described [19]. Plasmids encoding GST fusions of importin-α1, importin-α3, importin-α5, importin-α7 or importin-β were gifts from Dr. M. Köhler and have been previously described [20)]. Plasmid pGEX-6P Flag-Ran (Q69L) [21] and pGEX-2T SV40 NLS-G [22] were gifts from Dr. Y. Yoneda and have been described earlier. The other plasmids (File S1) were constructed using standard procedures [23]. The construction of plasmids pC.22K, and pC.22Kss (pC.22K containing substitutions in spice acceptor\donor nucleotide sequence without changing the amino acid sequence) is described elsewhere [8]. The protein expressed in plasmid pC.22K DNA transfected cells is recognized by 33K specific (anti-33Kp) sera but not by 22K specific sera (anti-22Kp) [8]. The protein expressed in plasmid pC.22Kss DNA transfected cells is recognized by 22K specific (anti-22Kp) sera but not by 33K specific sera (anti-33Kp) [8].

### Peptide synthesis and production of antisera

Production and characterization of antibody recognizing both 33K and 22K have been described [15]. To produce protein specific antiserum, two peptides (amino acid 141KLTKTATQSKKSRRSAS AARPRPPPLPPKRARAPRRPKGQRHQAD185 and 156ASAARPRPPPLPPKRAR APRRPKGQRHQADDASTEGRDKLRELIF200) representing 33Ks were synthesized on the Pioneer Peptide Synthesis system (Perkin Elmer) and conjugated to keyhole limpet hemocyanin (KLH) as a carrier molecule. Rabbits were immunized with conjugated peptide (500 µg/rabbit) emulsified with Freund's Complete Adjuvant (FCA) followed by two injections (conjugated peptide, 250 µg/rabbit) in Freund's incomplete adjuvant (FIA) at four weeks apart. Serum was collected twelve days after the third injection to test for protein specific antibodies.

For *in-vitro* nuclear import blocking experiment, nuclear import protein inhibitory peptides: importin-β binding (IBB) domain of importin-α [24], SR1 peptide [19], IBB rp L23a [25] and Ycbp80 [26], [27] were synthesized on the Pioneer Peptide Synthesis system (Perkin Elmer)

### Western blot analysis

Monolayers of MDBK or 293T cells were infected with wild-type BAdV-3 (MOI of 5) or transfected with individual plasmid DNA (2–5 µg/10^6^ cells), respectively. At indicated times post infection, the cells were collected and analyzed by Western blot as described [15] using protein-specific antisera.

### Recombinant protein expression and protein purification

Glutathione S-transferase (GST) and GST fusion proteins were purified using GST beads (GE Health care) as described [15] The recombinant proteins: GST-NLS-GFP and GST-RanQ69L [28], GST fused to importins-α1, -α3, -α-5, -α7 or –β1 [29] and GST-TRN-SR2 [19] were expressed in *E.coli* BL21 cells and purified as previously described [24], [26], [27], [30] The purified proteins were dialyzed using Slide-A-Lyzer dialysis cassette (Thermo scientific). The concentrations of the proteins were measured by Bradford assay (Bio Rad) using spectrophotometer (Pharmacia Biotech).

### *In-Vitro* nuclear import assay

MDBK cells at 80% confluency grown on cover slips were used in nuclear import assay as described earlier [31] with slight modifications [23]. The cells incubated with GST-NLS-GFP were visualized directly using Zeiss LSM 5 laser scanning con-focal microscope. However, the cells incubated with GST-33K were permeabilized with Triton X-100 and then stained with anti-33K serum and Cy3 conjugated goat anti-rabbit IgG before visualization.

### Immunofluorescence microscopy

The cells were infected with BAdV-3 or transfected with 1 µg individual plasmid DNA using lipofectamine as per manufacturers instructions (Invitrogen). At twenty hrs post infection and forty eight hours post-transfection, the cells were processed as described [23] and analyzed by confocal microscopy.

### GST pull down assay

Individual plasmid DNAs (0.8 µg) were used to synthesize radio-labeled wild-type or mutant 33Ks proteins and used in GST pull down assays as described [16].

### Construction of recombinant plasmid pFBAV33Ksr

A 237 bp DNA Fragment was amplified using primers SR1F (5′- AAATGGATCCTTAGGTTCA-3′) and SR1bam (5′- ATTCCAGCACACTGGCGGCCGTTA-3′) and pC.33K DNA as a template. A 229 bp DNA fragment was amplified by PCR using primers SRascI (5′-CGCCACTCAGAGCAAAAAGAGC-3′ and SR1R (5′-GCAGCTGCCCGTCA GTGAAC CTAAGGATCC-3′) and pC.33K DNA as a template. In third PCR reaction, the two PCR fragments that have 23 bp overlap were annealed and external primers SRascI and SR1bam were used to PCR across to give 462 bp amplicon. Next, a 279 bp DNA fragment was amplified by PCR using primers SR2F (5′-ATTCCAACAAAGTGGCGCTCAGCG-3′) and SR1bam, and 6299 bp DNA as a template. A 218 bp DNA fragment was amplified by PCR using primers SRascI and SR2R (5′-TTGAGGTGACAC CGCTG AGCGCCACTTTG-3′). The two PCR fragments that have 18 bp overlap were annealed and external primers SRascI and SR1bam were used to PCR across to give 462 bp amplicon. The 462 bp DNA fragment was digested with *Asc*I-*Bam*HI and ligated to 5911 bp *AscI*-*Bam*HI fragment of pC.33K creating plasmid pC.33Ksr.

A 5940 bp *Bam*HI fragment of plasmid pUC304A was isolated and ligated to 5515 bp *Bam*HI fragment of plasmid pC.3HA (unpublished) creating plasmid p33Kbam. A 11104 bp *Asc*I-*Bsp*EI fragment isolated from plasmid p33Kbam was ligated to 351 bp *Asc*I-*Bsp*EI fragment of plasmid pC.33Ksr creating plasmid p33KbamSR. The recombinant pFBAV33Ksr was generated by homologous recombination in *E. coli* between *Sfi*I linearized pPUC304A^+^ [17] and a 5940 bp *Bam*HI-*Psi*I fragment of plasmid p33KbamSR.

### Computer programs

All pictures have been generated using Power Point program included in Microsoft Office: Mac2011.

## Results

### Characterization of BAdV-3 33K protein

**Figure 1:**
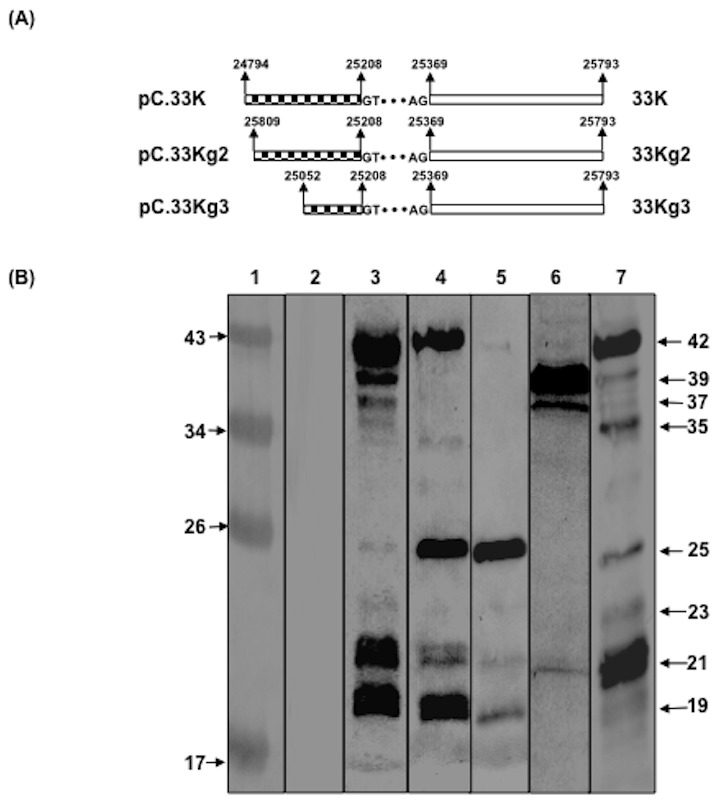
Analysis of BAdV-3 33K. (**A**). *Schematic representation of BAdV-3 33K*. The coding sequences shared by 33K and 22K (box with Pattern) or specific for 33K (hollow box) are depicted. The spliced region in 33K is represented by dots flanked by splice donor\acceptor sites. The wild-type (GT…AG) and mutated (GC…CG) splice acceptor/donor sites are depicted. The nucleotide numbers of BAdV-3 genome shown are according to Gene bank accession # AF030154 residues are underlined. The star represents the stop codon. The name of the encoded protein is depicted on the right of the panel. The name of the plasmid is depicted on the left of the panel. (**B**). *Western blot analysis*. Protein lysates of BAdV-3 infected MDBK cells or plasmid DNA transfected 293T cells were separated by 10% SDS-PAGE, transferred to nitrocellulose membrane and probed with anti-33Kp serum. The position of the molecular weight markers (M) in kDa is shown to the left of the panel. Arrows on the right of the panel indicate the position of the identified protein in kDa.

Earlier, we demonstrated that spliced (33K) and unspliced (22K) forms of 33K protein are encoded by L6 region of BAdV-3, which have unique C-terminal region but share a N-terminal 138 amino acids [8]. In order to characterize the 33K (spliced) protein, specific antibodies against peptides representing the unique C-terminal region of predicted 33K (anti-33Kp serum) protein were generated and used in Western blot using plasmid DNA (Fig. 1B) transfected 293T cells and BAdV-3 infected MDBK cells. Anti-33Kp serum detected five major proteins of 42 kDa, 39 kDa, 37 kDa, 21 kDa and 19 kDa, and three minor proteins of 35 kDa, 25 kDa and 23 kDa in BAdV-3 infected MDBK cells at 48 hrs post infection (Fig. 1B, lane 3). Like in BAdV-3 infected cells, similar protein bands (different intensities) are also detected in 293T cells (Fig. 1B, lane 7) transfected with plasmid pC.33K expressing 33K protein (Fig. 1A). No such protein could be detected in mock infected MDBK cells (Fig. 1B, lane 1).

**Figure 2:**
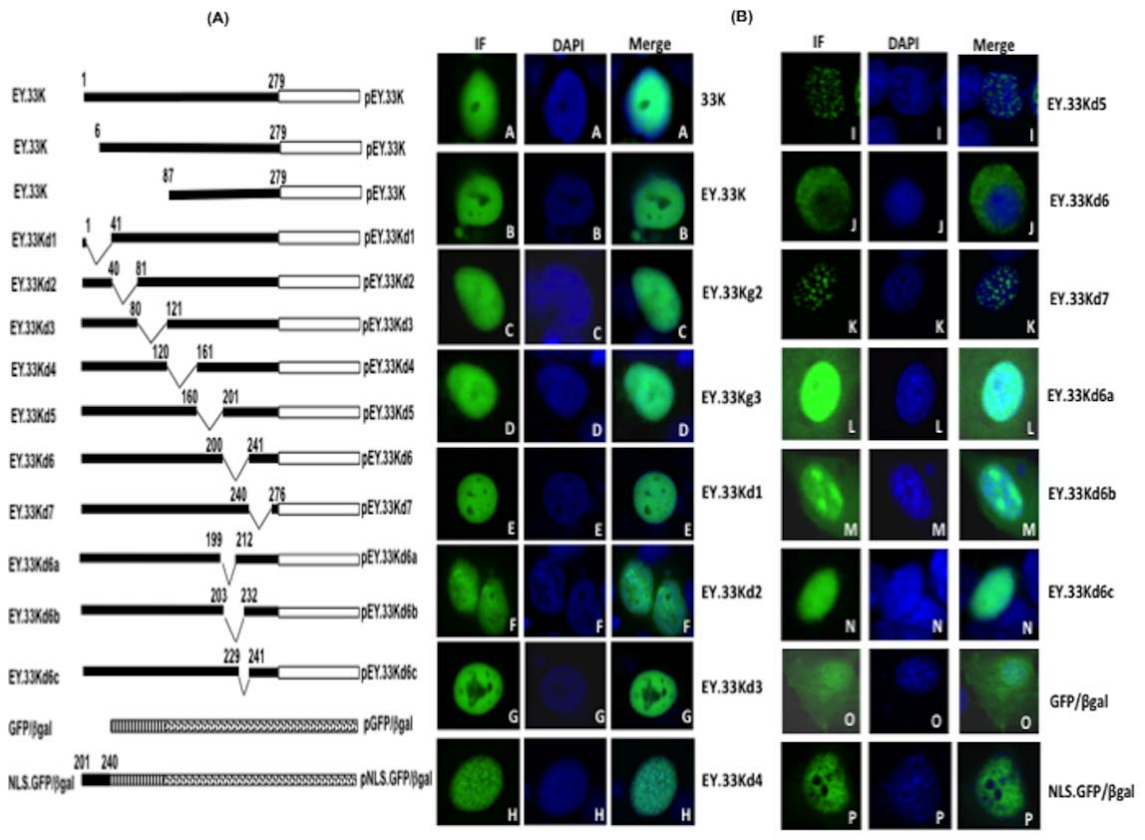
Sub cellular localization of 33K proteins. (**A**) *Schematic representation of BAdV-3 33K fusion proteins*. The number above the box denote the amino acid number for 33K protein. Thick box represent BAdV-3 DNA; hollow box represents EYFP DNA; stripped box represents GFP DNA; dotted box represents β-gal DNA. Thin lines represent deleted regions. The name of the individual mutant protein is indicated on the left of the panel. The name of the individual plasmid is indicated on the right of the panel. (**B**). *Immunofluorescence*. Monolayers of MDBK cells (panel A) infected with BAdV-3. At 24 hrs post infection, the cells were analysed by indirect immunofluorescence by staining with anti-33Kp serum, Cy 2 conjugated goat anti-rabbit serum and DAPI. Similarly, monolayers of HeLa cells (panels B to P) were transfected with individual plasmid DNA expressing indicated 33K fusion proteins and analysed at 48 hrs post transfection. Finally, the cells were stained with DAPI and analyzed by direct fluorescence. The name of the protein is indicated on the right of the panel.

Anti-33Kp serum detected four major proteins of 42 kDa, 25 kDa, 21 kDa and 19 kDa in 293T cells transfected (Fig. 1B, lane 4) with plasmid pC.33Kg2 DNA (Fig. 1A). However, only three proteins of 25 kDa, 21 kDa and 19 kDa (Fig. 1B, lane 5) could be detected in 293T cells transfected with plasmid pC.33Kg3 DNA (Fig. 1A). Similarly, three major proteins of 39 kDa, 37 kDa and 21 kDa (Fig. 1B, lane 6) could be detected in 293T cells transfected with plasmid pC.33Kd6b DNA (Fig.2A).

### Nuclear localization of 33K

Earlier, using anti-33K serum (which recognizes both 33K and 22K) we demonstrated that 33K/22K proteins localizes to the nucleus of BAdV-3 infected cells [15]. To determine if 33K protein is transported to the nucleus of the cells, MDBK cells were infected with wild-type BAdV-3 and examined by confocal microscopy using anti-33Kp serum. As seen in Fig. 2B, 33K (panel A) was predominantly localized in the nucleus of the infected cells. To determine if 33K protein is transported to the nucleus in the absence of other viral proteins, we analyzed plasmid pEY.33K DNA (Fig. 2A) transfected HeLa cells by con-focal microscopy. Like BAdV-3 infected cells, EY.33K protein (Fig. 2A) was predominantly localized in the nucleus of the transfected cells (Fig. 2B, panel B). Similarly, EY.33Kg2 protein (Fig. 2A) initiated at 2^nd^ methionine residue (amino acid 6) (Fig. 2B, panel C) or EY.33Kg3 protein (Fig. 2A) initiated at third methionine residue (amino acid 87) (Fig. 2B, panel D) predominantly localized to the nucleus of transfected cells (Fig. 2B).

To determine the domain(s) responsible for nuclear localization of 33K protein, a panel of plasmids encoding mutant proteins were constructed (Fig. 2A). These deletions were confirmed by restriction enzyme analysis and sequencing of mutant plasmid DNA. HeLa cells were transfected with individual mutant plasmid DNAs and analyzed at 48 hrs post-transfection by confocal microscopy (Fig. 2B). As seen in Fig. 2B, the deletion of amino acids 2–40 (EY.33Kd1)(panel E), 41–80 (EY33Kd2) (panel F), 81–120 (EY.33Kd3) (panel G) and 120–160 (EY.33Kd4) (panel H) did not affect nuclear localization (Fig. 2B). Similarly, mutant 33K proteins lacking amino acid 161–200 (EY33Kd5) (panel I) and 241–275 (EY33Kd7) (panel K) also localized to the nucleus. However, compared to 33K (panel B), mutant EY.33Kd5 (panel I) and EY33Kd7 (panel K) showed granular distribution within the nucleus. In contrast, mutant EY.33Kd6 lacking amino acid 201–240 localized predominantly in the cytoplasm (panel J). Taken together these results indicate that amino acid 201–240 contain NLS for nuclear localization of BAdV-3 33K protein (Fig 2B).

To further define the NLS, we constructed plasmids containing smaller deletions in this region of 33K (Fig. 2A) and analyzed the localization of mutant proteins in transfected cells using confocal microscopy. None of these deletions (Fig. 2B, panels L, N) localized predominantly in the cytoplasm of transfected cells. However, mutant EY.33Kd6b protein (Fig. 2A) containing deletion of amino acid 204–231 localized predominantly in the nucleolus of the transfected cells (Fig. 2B, panel M). These results suggest that amino acid 201–240 of 33K may contain multiple NLSs. Analysis of the amino acid sequence of BAdV-3 33K protein containing putative NLS did not reveal stretches of basic amino acid residues that resemble the classical NLS. To determine if amino acids 201–240 contain NLS, we fused this domain to chimeric GFP/β-gal protein (Fig. 2A) [32]. As expected [32], the chimeric GFP/β-gal protein (Fig. 2A) was predominantly located in the cytoplasm (Fig. 2B, panel O). However, the chimeric GFP/β-gal protein fused to 33K NLS domain (NLS.GFP/β-gal; Fig. 2A) was predominantly localized to the nucleus of the transfected cells (Fig. 2B, panel P). These results confirm that amino acid 201–240 contains essential element(s) of the NLS of BAdV-3 33K protein.

### Nuclear localization of mutant 33Ksr

**Figure 3:**
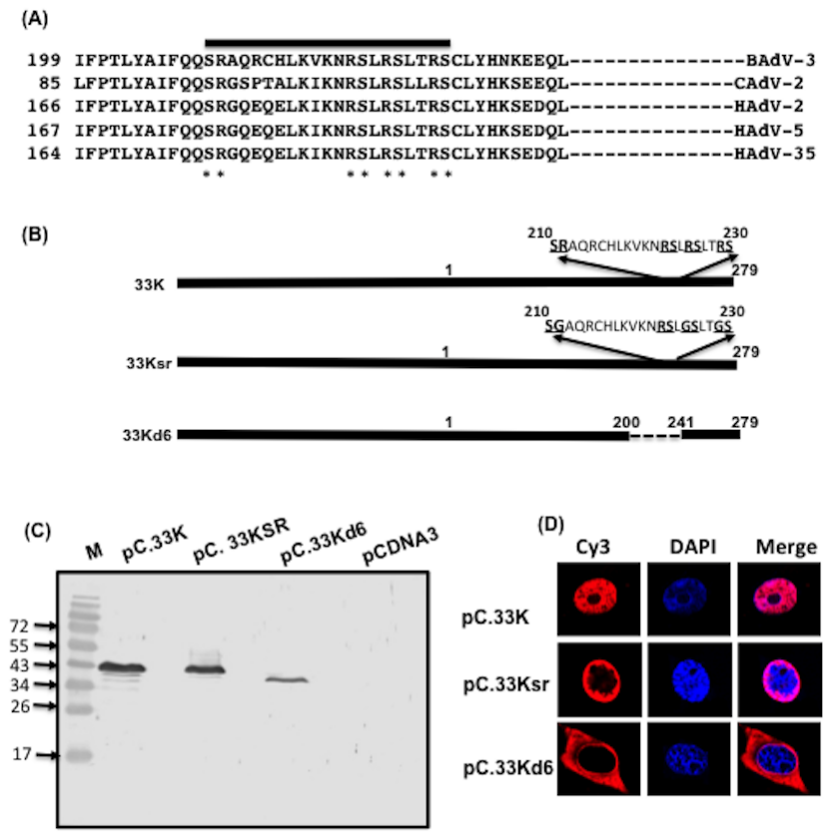
Sub cellular localization of mutant 33Ksr protein. (**A**) *Amino acid homology of BAdV-3 33K like proteins*. Alignment of deduced amino acid sequences of BAdV-3 33K homologs with those of canine adenovirus (CAdV)-2 (GeneBank Accession # AC_000020), HAdV-2 (GeneBank Accession # AC_000007), HAdV-5 (GeneBank Accession # AC_000008), HAdV-35 (GeneBank Accession # AC_000019). The potential RS domain is overlined. RS repeat residues are indicated (*). (**B**) *Schematic representation of BAdV-3 33K fusion proteins*. The number above the thick box denote the amino acid number for 33K protein. Thick box represent BAdV-3 DNA. Dotted lines represent deleted regions. The name of the individual mutant protein is indicated to the left of the panel. The name of the individual plasmid is indicated on the right of the panel. (**C**) *Western blot*. Proteins from lysates of 293T cells transfected with indicated plasmids were separated by 15% SDS-PAGE, transferred to nitrocellulose and probed in Western Blot using anti-33K serum [15]. (**D**) *Immunofluorescence*. Monolayers of HeLa cells were transfected with indicated individual plasmid DNA and analysed at 48 hrs post transfection by indirect immunofluorescence by staining with anti-33Kp serum [15], Cy 3 conjugated goat anti-rabbit serum and DAPI and analyzed by direct fluorescence. The name of the plasmid is indicated on the left of the panel.

Analysis of amino acid 201–240 of BAdV-3 33K identified a RS repeat at amino acids 210–230, which appeared conserved among different members of *Mastadenovirus* (Fig. 3A). Since RS repeats are involved in the transport of proteins to the nucleus [33], we constructed plasmid pC.33Ksr (Fig. 3B) containing substitution of arginines at position 211, 225 and 229 with glycines and analyzed the expression (Fig. 3C) and localization (Fig. 3D) of the mutant 33K protein in transfected cells. As seen in Fig. 3D, 33K localizes to the nucleus while mutant 33Kd6 localize predominantly to the cytoplasm of the transfected cells. Interestingly, mutant 33Ksr protein also localizes to the cytoplasm in transfected cells.

### Nuclear import of 33K

**Figure 4:**
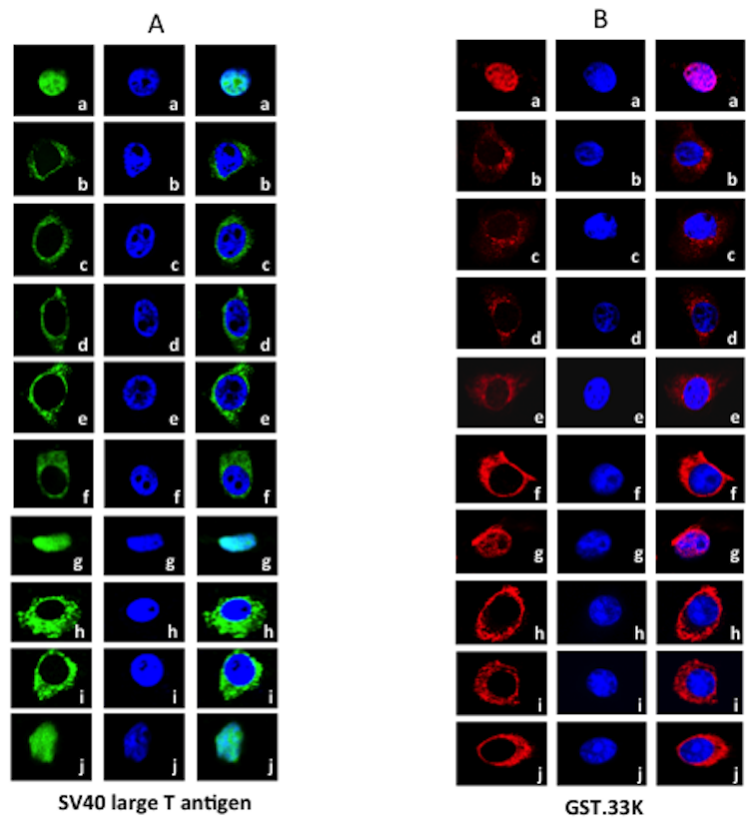
*In-vitro* nuclear import assays. MDBK cells were permeabilized with digitonin and incubated with (**A**) GST-NLS-GFP (panels a,b,c,d,e,f,g,h,I,j) or (**B**) GST-33K (panels a,b,c,d,e,f,g,h,I,j). Import reactions were carried out in the presence (panel a) or absence of rabbit reticulocyte lysates (panel b), absence of ATP generating system (panel c), in the presence of the dominant negative mutant RanQ69L (panel d), in the presence of wheat germ agglutinin (WGA) (panel e), incubation at 40C (panels f), in the presence of inhibitory peptides IBB Impα (panel h), IBBrpL23α (panel g) Ycbp80 (panels i), SR1 (panel j).

To determine the requirements for nuclear transport of 33K, we established an *in vitro* nuclear import assay in digitonin permeabilized cells. First, we determined the integrity of the assay by analyzing the nuclear import of GST-NLS-GFP (containing SV40 LT NLS) fusion protein containing classical importin-α/-β dependent NLS by direct fluorescence (Fig. 4A). As expected, the nuclear import of GST-NLS-GFP a) required cytosolic factors (panels a, b) and ATP (panel c). Moreover, the nuclear import appeared temperature sensitive (panel f) and was inhibited the presence of wheat germ agglutinin (panel e) or RanQ69L (ran mutant deficient in hydrolysis of ATP) (panel d).

Next, we analyzed the nuclear import of 33K in digitonin permeabilized cells by indirect immunofluorescence using anti-33Kp serum (Fig. 4B). The 33K protein was efficiently imported into the nuclei in the presence (panel a) but not in the absence of rabbit reticulocyte lysate (panel b) or ATP regenerating system (panel c). Addition of RanQ69L protein (panel d) or wheat germ agglutinin (panel e) or completely inhibited the nuclear transport of 33K. Moreover, nuclear import was inhibited at 4°C in the presence of cytosolic factors (panel f). These results suggested that nuclear import of 33K occurs through the nuclear pore complex and is mediated by soluble factors/receptors.

### Nuclear import of 33K in the presence of peptide inhibitors

The soluble factors which mediate nuclear import of proteins include importin-α, importin-β and \or transportin. To determine the nature of soluble factor(s), which mediate nuclear transport of 33K initially, we used different peptides, which are known to block the individual soluble factor specific nuclear transport in digitonin permeabilized cells. A 41amino acid specific peptide representing importin-β binding (IBB) domain of importin-α [24] or a 30 amino acid peptide representing importin-α binding domain of Ycbp80 protein [26], [27] act as inhibitor of importin-α mediated import. A 32 amino acid peptide representing importin-β binding domain of ribosomal protein rpL23a acts as inhibitor of importin-β mediated import [25]. A 29 amino acid peptide (SR1) containing eight RS repeats flanked by two arginine rich stretches acts as inhibitor of transportin-3 mediated import [19]. As expected (Fig. 4A) addition of Ycbp80 peptide (panel i) or IBB domain peptide (panel h) to reticulocyte lysate in permeabilized cells inhibited import of GST-NLS-GFP protein. Moreover, addition of either rpL23a peptide (panel g) or SR1 peptide (panel j) to reticulocyte lysate in permeabilized cells did not block the nuclear import of GST-NLS-GFP protein. Similarly (Fig. 4B), addition of Ycbp80 peptide (panel i) or IBB domain peptide (panel h) but not rpL23a peptide (panel g) inhibited the nuclear localization of GST-33K. Interestingly, addition of SR1 peptide also inhibited the nuclear import of GST-33K (panel j). This suggested that nuclear transport of 33K utilizes both importin-α and transportin-3 receptor dependent pathways.

### Interaction of 33K with importin receptors

**Figure 5:**
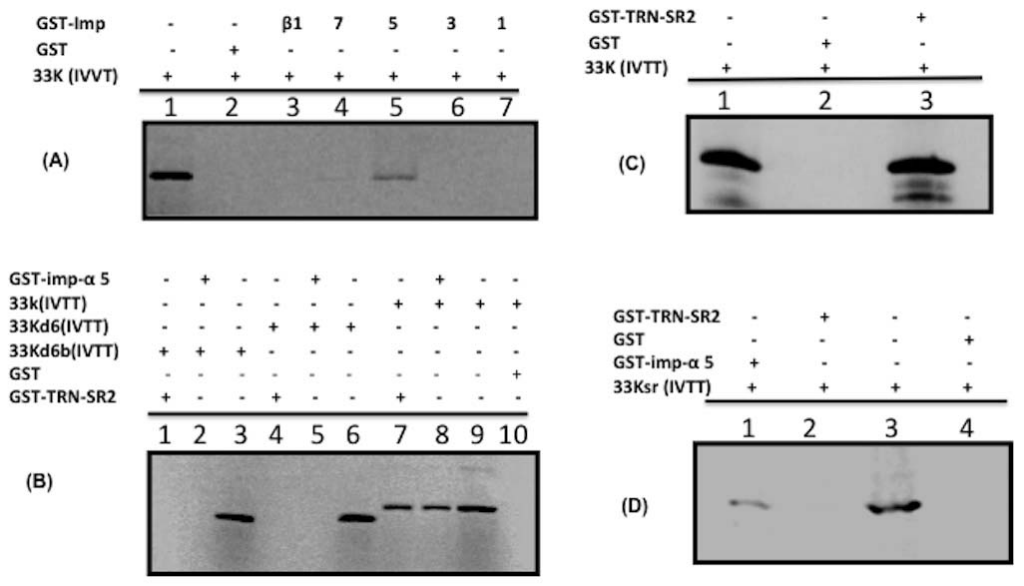
*In-vitro* interaction of 33K with transport receptors. (**A**) Purified GST fusions of importin -α1 (lane 7), -α3 (lane 6), -α5 (lane 5), -α7 (lane 4) or importin -β1 (lane 2) along with GST alone were incubated with *in -vitro* transcribed and translated, [^35^S]-labeled 33K. Input [^35^S]-labeled 33K (lane 1). (**B**) Purified GST fusions of importin-α5 (lanes 2,5,8) or TRN-SR2 (lanes 1,4,7) along with GST alone (lane 10) were incubated with *in-vitro* transcribed and translated [^35^S]-labeled 33K (lanes 8,10), 33Kd6 (lanes 4,5) or 33Kd6b proteins (lanes 1,2). Input [^35^S]-labeled 33K (lane 9), [^35^S]-labeled 33Kd6 (lane 6), [^35^S]-labeled 33Kd6b (lane 3) (**C**) Purified GST-TRN-SR2 fusion protein (lanes 2,3) or GST alone (lane 2) were incubated with *in-vitro* transcribed and translated [^35^S]-labeled 33K (lane 2,3). Input [^35^S]-labeled 33K (lane 1). (**D**) Purified GST- importin-α5 fusion protein (lane 1), GST-TRN-SR2 fusion protein (lane 2) or GST alone (lane 4) were incubated with *in-vitro* transcribed and translated [^35^S]-labeled 33Ksr protein (lanes1, 3). Input [^35^S]-labeled 33K (lane 3). Samples from (A), (B), (C) and (D) were pulled down with glutathione Sepharose beads, separated by 10% SDS-PAGE and visualized using a phosphor screen. 10% of the input [^35^S]-protein was run as a control.

Six different forms of importin-α have been identified in mammalian cells [34]. To determine the interaction between 33K protein and import receptors, we performed GST-pull down assay using purified GST alone or GST fusion proteins of importin-α1, importin-α3, importin-α5, impotin-α7 and importin-β1 individually immobilized on glutathione-Sepharose beads (Pharmacia) with *in vitro* translated [^35^S] methionine labeled 33K protein. The bound proteins were separated by 10% SDS-PAGE and analyzed by autoradiography. As seen in Fig. 5A, GST-α5 fusion protein was able to bind radiolabelled 33K protein (lane 5) as similar protein was observed in input protein control (lane 1). No such binding was observed when purified GST alone (lane 2) or GST fusions of importin-α1 (lane 7), importin-α3 lane 6), importin-α7 (lane 4) or importin-β1 (lane 3) bound to glutathione sepharose beads were used in pull down assays.

To determine the domain involved in binding to importin-α5, GST-α5 fusion protein bound to glutathione sepharose beads was used in GST pull down assay using radiolabelled mutant 33K proteins. As seen in Fig. 5B, GST-α5 was able to bind radiolabelled 33K protein (lane 8). Radiolabelled 33K was used as input protein (lane 9). No such protein bound to GST alone (lane 10). Similarly, no such binding was observed when radiolabelled mutant 33K containing deletion of amino acid 201-240 (33Kd6) (lane 5) was used in the GST pull down assay. Radiolabelled 33Kd6 alone (lane 6) was used as input protein. Similarly, no such binding was observed when radiolabelled mutant 33Ks containing deletion of amino acid 203-232 (33Kd6b) (lane 2) was used in the GST pull down assay. Radiolabelled 33Kd6b alone (lane 3) was used as input protein.

### The interaction of 33K with transportin-3

To determine if 33K also interacts with transportin-3, GST pull down assay was performed using GST alone or GST-transportin fusion protein and *in vitro* translated [^35^S] methionine labeled 33K protein. As seen in Fig. 5, GST-TRN-SR2 (transportin -3) immobilized on glutathione-Sepharose beads were able to bind [^35^S] methionine labeled 33K protein (panel B, lane 7; panel C, lane 3). Radiolabelled 33K was used as input protein (panel C, lane 1). No such binding was observed when purified GST bound to glutathione-Sepharose beads were used in pull down assay (panel C, lane 2). To determine the domain involved in binding to transportin-3, GST-TRN-SR2 fusion protein bound to glutathione Sepharose beads was used in GST pull down assay using radiolabelled 33K proteins. As seen in Fig. 5B, GST-TRN-SR2 was able to bind radiolabelled 33K protein (lane 7). Radiolabelled 33K protein was used as input sample (lane 9). Similarly, no such binding was observed when radiolabelled mutant 33Ks containing deletion of amino acid 201-240 (33Kd6) (lane 4) was used in the GST pull down assay. Radiolabelled 33Kd6 protein alone (lane 6) was used as input sample. Similarly, no such binding was observed when radiolabelled mutant 33Ks containing deletion of amino acid 203-232 (33Kd6b) (lane 1) was used in the GST pull down assay. Radiolabelled 33Kd6b alone (lane 3) was used as input protein.

To determine if RS motif (Fig. 3A) is involved in binding to transport receptors, we constructed plasmid pC.33Ksr expressing mutant 33Ksr protein (Fig. 3B) in which arginines at amino acid 211, 225 and 229 in RS motif were replaced with glycine residues. As seen in Fig. 5D, GST-α5 fusion protein bound to glutathione Sepharose beads was able to bind radiolabelled 33Ksr (lanes 1). Radiolabelled 33Ksr protein was used as input sample (lane 3). No such binding was observed when purified GST bound to glutathione-Sepharose beads (lane 4) or GST-TRN-SR2 (lane 2) was used to pull down radiolabelled 33Ksr protein.

### Isolation of mutant BAV33Ksr

**Figure 6:**
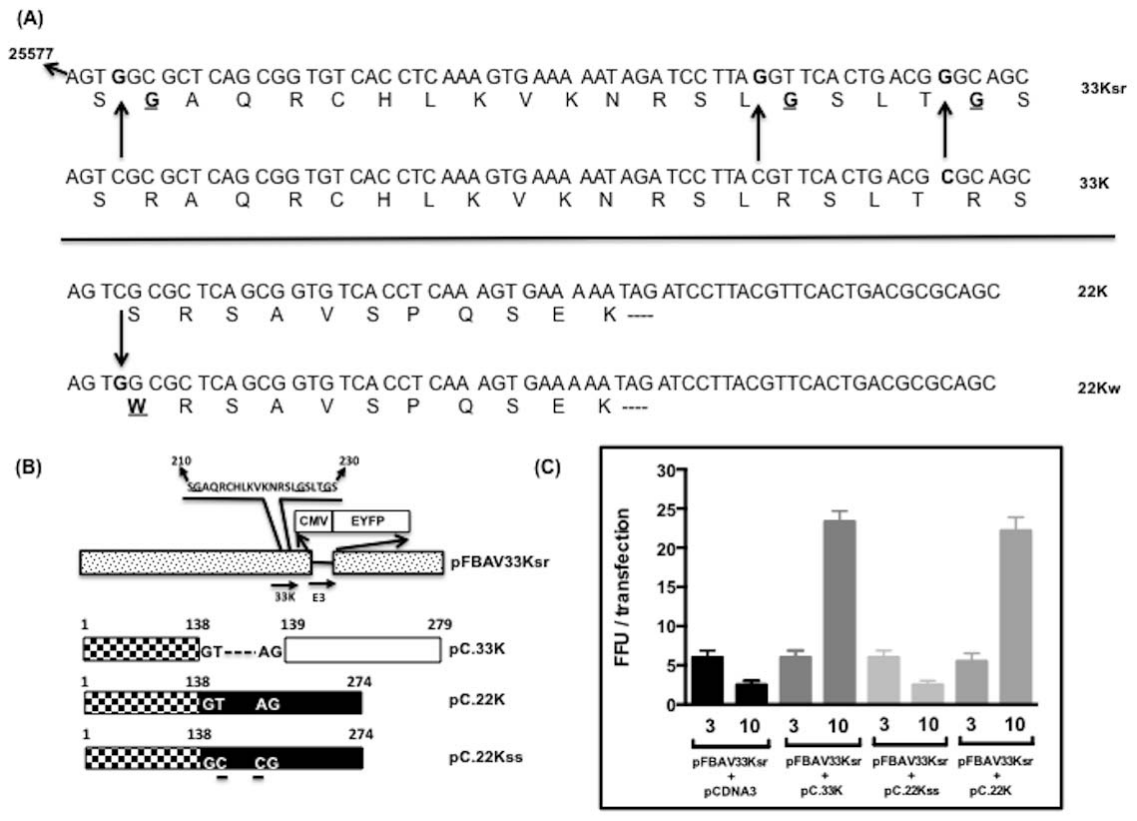
L6 33K and 22K. (**A**) *Sequence of 33K and 22K*. The nucleotide numbers of BAdV-3 genome shown are according to Gene bank accession # AF030154. The changed nucleotide residues in 33K and 22K are shown in bold. The substituted amino acid in 33Ksr or 22Kw is shown in bold and underlines. (**B**). *Schematic representation of plasmid pFBAV33Ksr*. BAdV-3 genome (dotted pattern box). The thin line represented deleted E3 region [45]. The substituted amino acids (glycines) of 33K in BAdV-3 genome are underlined. The numbers represent the amino acid numbers of 33K. CMV (human cytomegalovirus immediate early promoter); EYFP (enhanced yellow fluorescent protein). Arrows represent the direction of transcription. The coding sequences shared by 33K and 22K (box with pattern) or specific for 33K (hollow box) and 22K (black box) are depicted. The spliced region in 33K is represented by dots flanked by splice donor\acceptor sites. The wild-type (GT…AG) and mutated (GC…CG) splice acceptor/donor sites are depicted. The numbers of the top denote amino acid numbers. (**C**) *Complementation of pFBAV33Ksr genome*. The VIDO DT1 cells were co transfected with indicated plasmids and the fluorescent focus forming units were counted at indicated days post transfection. The numbers on the X- axis denote the days post transfection.

To determine if RS motif of 33K is essential for the replication of BAdV-3, we successfully substituted arginines with glycines at amino acid 211, 225 and 229 of 33K. These changes also substituted serine with tryptophan at amino acid 264 of 22K (Fig. 6A). Taking advantage of homologous recombination machinery of *Escherichia coli* [35], we constructed full plasmid pFBAV33Ksr containing glycines at amino acid 211, 225 and 229 of BAdV-3 33K (Fig. 6B). The plasmid pFBAV33Ksr DNA was transfected into non complementing VIDO DT1 cells. Appearance of cytopathic effects in plasmid BAdV-3 genomic DNA transfected cells takes a minimum of 5–10 days [17]. Absence of appearance of cytopathic effects \fluorescent cells even after 20 days post transfection suggested the absence of formation of any progeny virus. Co-transfection of VIDO DT1 cells with plasmid pFBAV33Ksr and pcDNA3 (Invitrogen) DNAs also did not produce any progeny virus. In contrast, co- transfection of VIDO DT1 cells with plasmid pFBAV33Ksr and pC.33K (express protein recognized by anti-33Kp) DNAs or plasmid pFBAV33Ksr and pC.22K (express protein recognized by anti-33Kp but not anti-22Kp) DNAs produced progeny virus as indicated by production of cytopathic effects\fluorescent cells. However, co-transfection of plasmid pFBAV33Ksr and pC.22Kss (express protein recognized by anti-22Kp) DNAs did not produce cytopathic effects\fluorescent cells. The fluorescent focus-forming units per field were used to count total FFU in a well under fluorescent microscope (Fig. 6C).

## Discussion

The L6 region of BAdV-3 encodes non-structural (33K and 100K) and structural (pVIII) proteins [7]. Earlier, we reported that BAdV-3 33K detected as three proteins of 42, 38 and 33kDa in infected cells appear to be required for capsid assembly and efficient DNA capsid interaction [15]. However, recent report suggests that 33K and 22K proteins are produced from spliced and unspliced forms of L4 transcripts of HAdV-2, respectively [12]. Since little is known about the existence of different forms of 33K in BAdV-3 infected cell, we sought to analyze this in detail. Here, we report further characterization, cellular distribution and putative pathways mediating nuclear transport of BAdV-3 33K protein.

Analysis of our earlier data [15] suggested that antisera, generated against C-terminus 197 amino acids of putative 22K protein could recognize both 33K and 22K proteins in BAdV-3 infected cells. Thus, reduced formation of mature virions in mutant BAdV-3 infected cells [15] could be due to inactivation of both 33K and 22K proteins. Although 33K mRNA is predicted to encode a protein of 279 amino acids, 33K specific antisera detected five major proteins of 42 kDa, 39 kDa, 37 kDa, 21 kDa and 19 kDa, and three minor proteins of 35 kDa, 25 kDa and 23 kDa. It is possible that different forms of 33K are generated by different mechanisms including translation from different ATG codons and by alternate splicing. Analysis of our data suggest that 25 kDa protein appears to be translated from third ATG (amino acid 87). Similarly, 39 kDa protein appears to be generated by alternative splicing. Although we have not detected such mRNA in BAdV-3 infected cells, such rare mRNA has been detected in HAdV-5 infected cells [37].

The 33K proteins are detected predominantly in the nucleus of BAdV-3 infected cells. Although proteins less than 40 kDa in size can diffuse passively into the nucleus [37], it is unlikely that BAdV-3 33K protein enters nucleus by simple diffusion mechanisms. Support for this comes from the fact that EYFP (a cytoplasmic protein) fused to 33K is predominantly localized to the nucleus of infected cells. Secondly, nuclear transport by diffusion mechanism is expected to result in equal distribution of the protein throughout the cell rather than accumulating in the nucleus. Thirdly, results of an *in-vitro* nuclear import assay using rabbit reticulocyte lysate suggest that nuclear import of 33K utilizes a Ran-dependent pathway requiring energy and ATP, is receptor mediated and involves nuclear pores.

Since known nuclear import pathways including transportin and importin-α/-β mediated pathways are functional in our *in-vitro* nuclear import assay, we determined the importance of each import pathway using inhibitory peptides in nuclear import assay using digitonin permeabilized cells, and GST pull down assays. Our data demonstrate that nuclear import of 33K is facilitated by transportin-3 of transportin pathway and importin-α5 of import-α/-β pathways. Similar results have been reported earlier for nuclear import of adenovirus pVII protein, which also uses multiple nuclear import receptor pathways [4], [5].

Initial analysis of mutant BAdV-3 33K proteins demonstrated that amino acid 201–240 contain potential NLS(s), which was sufficient to transport predominantly cytoplasmic GFP/β-galactosidase fusion protein to the nucleus of the cell. Consistent with our localization results, analysis of interaction of 33K and mutant 33kd6 proteins with GST- importin-α5 or GST-transportin-3 fusion proteins also identified amino acid 201–240 in BAdV-3 33K involved in these protein interactions. These results provide convincing evidence that amino acid 201–240 is involved in nuclear localization of 33K. However, further analysis of 33K mutants containing smaller deletions in the identified domain (amino acid 201–240) suggested that 33K may contain multiple and/or overlapping NLS in this region, which may be involved in binding importin-α5 and transportin-3.

Peptide inhibition and protein binding assays suggest that similar or overlapping regions of a 33K protein appear to act as recognition sites for both importin-α5 and transportin-3. Interestingly, 33K protein (amino acid 201–240) contains a pseudo RS domain (three RS motifs and one SR motif; Fig. 5), which is conserved in different adenoviruses including HAdV-5 33K and is suggested to be involved in activating adenovirus IIIa RNA splicing [36]. The RS domain of proteins has been shown to be involved in splicing and nuclear localization of SR proteins [38], [39], [40]. The nuclear transport of SR protein is mediated by interaction of specific RS domain with nuclear import receptor transportin-3 [19]. Earlier report suggested that serine residues of RS motif of HAdV-5 33K are important for nuclear localization in transfected cells [41].

Several evidences support the observation that the RS domain (Fig. 5) is responsible for the transportin -3 mediated nuclear localization of BAdV-3 33K protein. First, RS domain deleted (amino acid 204–231) 33K is not located predominantly in the nucleus of the cell. Secondly, 33K protein interacts with SR protein nuclear import receptor transportin-3. Thirdly, the nuclear import of 33K is inhibited by transportin-3 mediated nuclear import inhibitor SR1 peptide [19]. Finally, disrupting RS motif (mutation of arginine residues to glycine) abolishes the binding of transportin-3 to mutant 33K protein but has no effect on binding of 33K to importin-α5.

A number of viral proteins including HIV Rev utilize multiple nuclear import receptors for nuclear accumulation [4], [42]. Our results suggest that 33K utilizes multiple nuclear import pathways. It is possible that multiple pathways provide redundancy, which increase the efficiency of nuclear transport of 33K. Alternatively, it is possible that abundance [42] and affinity of the receptors for 33K determine the choice of pathway used for nuclear import of 33K.

Analysis of 33K deleted HAdV-5 suggested that 33K is essential for replication of HAdV-5 including encapsidation of viral genome [44]. Earlier, analysis of a mutant HAdV-5 expressing C-terminal truncated 33K, which did not disrupt 22K reading frame suggested that C-terminal 47 amino acids of 33K are essential for virus replication [43]. Analysis of C-terminal 47 amino acids suggest that it contains conserved RS dipeptides (Fig. 3A). *In -vitro* analysis of 33K mutants containing the substitution of serines with glycines suggested that RS dipeptides are essential for efficient splicing of IIIa mRNAs [36]. Although levels of IIIa mRNA were drastically reduced in 33K deleted HAdV-5 infected cells, the levels of most late viral proteins including formation of empty capsids was not effected suggesting that splicing enhancing activity of RS dipeptides of 33K (36) does not appear essential for the late gene expression and capsid formation [14].

The substitution of arginines with glycines at positions 211, 225 and 229 of BAdV-3 33K resulted in inhibiting the production of progeny virus. This could be due to the loss of binding to transportin-3 receptor and localization of BAdV-3 33K predominantly to the periphery of the nucleus. The altered localization of 33K could affect the function of the protein in the nucleus i. e late viral gene expression or viral DNA packaging [14], [15]. Alternatively, it is possible that arginine residues at 211, 225 and 229 could be involved directly in protein - DNA interactions leading to packaging of viral DNA by an unknown mechanism. It would be interesting to isolate and characterize BAdV-3 33K mutant (BAV33Ksr) by developing complementing cell lines.

The substitution of arginine with glycine at amino acid 211 of 33K also resulted in substitution of serine to tryptophan at amino acid 264 of 22K (22Kw: Fig. 6A). However, providing 22K in trans by co -transfection of plasmid pFBAV33Ksr with pC.33Kss (Fig. 6B) did not produce any cytopathic effect (Fig. 6C) suggesting that substitution of serine to tryptophan at amino acid 264 did not affect the production of progeny virions in plasmid pFBAV33Ksr DNA transfected cells. Moreover, earlier report suggested that non conserved C-terminal 23 amino acids of HAdV-5 22K are not essential for its function [44].

## Supporting Information

File S1 (DOCX)
