# Supplementary material for: Conserved Arginines of Bovine Adenovirus-3 33K Protein Are Important for Transportin-3 Mediated Transport and Virus Replication
Source: PLoS One. 2014 Jul 14;9(7):e101216. doi: 10.1371/journal.pone.0101216 (PMC4096500; doi:10.1371/journal.pone.0101216)
Supplement: File S1 — (DOCX) [file pone.0101216.s001.docx]

***File S1***

**CONSERVED ARGININES OF RS MOTIF OF BOVINE ADENOVIRUS-3 33K PROTEIN ARE IMPORTANT FOR TRANSPORTIN -3 MEDIATED NUCLEAR TRANSPORT AND VIRUS REPLICATION**

***a) Plasmids pC.33K, pC.33Kg2 and pC.33Kg3***. i) A **845 bp *NdeI-EcoRI* fragment of** plasmid pGEX.33K was isolated, blunt end repaired with T4 polymerase and ligated to *Hind*III digested (blunt end repaired with T4 polymerase) pCDNA3 (Invitrogen), creating the plasmid pC.33K (Fig. 1A). ii) A 862 bp *NdeI-AscI* fragment of plasmid pEY.33Kg2 was isolated and ligated to 5.4 kb *Nde*I-*Asc*I digested plasmid pC.33K creating plasmid pC.33Kg2 Fig. 1A). iii) A **649 bp *Aat*II*-Eco*RI fragment of** plasmid pC.33K was isolated, blunt end repaired with T4 polymerase and ligated to *Hind*III digested (blunt end repaired with T4 polymerase) plasmid pcDNA3, creating plasmid pC.33Kg3 (Fig. 1A).

***b) Plasmids pEY.33K, pEY.33Kg2, pEY.33Kg3***. i) A 845 bp fragment containing 33K ORF (spliced) was amplified by PCR [using primers P1 and P4 (Table 1), and plasmid pGEX.33K DNA as a template], digested with *Eco*RI-*Bam*HI and ligated to *Eco*RI-*Bam*HI digested plasmid pEYFP-N1 (Clontech) creating plasmid pEY.33K (Fig. 2A). ii) A 825bp fragment containing 33K ORF (spliced) initiating from 2^nd^ ATG was amplified by PCR [using primers P2 and P4 (Table 1), and plasmid pGEX.33Ks DNA as template], digested with *Eco*RI-*Bam*HI and ligated to *Eco*RI-*Bam*HI digested pEYFP-N1 creating plasmid pEY.33Kg2 (Fig. 2A). iii) A 525bp fragment containing 33K ORF (spliced) initiating from 3^rd^ ATG was amplified by PCR [using primers P3 and P4 (Table 1) and plasmid pGEX.33Ks DNA as template], digested with *Eco*RI-*Bam*HI and ligated to *Eco*RI-*Bam*HI digested pEYFP-N1 creating plasmid pEY.33Kg3 (Fig. 2A).

***c) Plasmids expressing EYFP-33K deletions33K deletion****. i) pEY.33Kd1.* A 220 bp fragment was amplified from by PCR using the primers P5 and P6 (Table 1) and plasmid EY.33K DNA as a template. Similarly, a 725 bp fragment was amplified by PCR using the primers P4 and P7 (Table 1) and plasmid pEY.33K DNA as a template. In a third PCR reaction, two PCR fragments that have 9 bp of internal overlap were annealed and external primers P4 and P8 (Table 1) were used to PCR across to give a 765 bp amplicon. This PCR product was digested with *Eco*RI-*Bam*HI and ligated to *Eco*RI-*Bam*HI digested pEYFP-N1 creating plasmid pEY.33Kd1 (Fig. 2A)*. ii) pEY.33Kd2.* A 440 bp *Sac*II-*Asc*I fragment of plasmid p33Kd2 containing deletion of amino acid 41-80 was isolated and ligated to 5.4 kb *Sac*II-*Asc*I fragment of plasmid pEY.33K creating plasmid pEY.33Kd2 (41-80 amino acid deletion). *iii) pEY.33Kd3.* A 440 bp *Sac*II-*Asc*I fragment of plasmid p33Kd3 (Kulshrestha and Tikoo, 2008 ) containing deletion of amino acid 81-120 was isolated and ligated to 5.4 kb *Sac*II-*Asc*I fragment of plasmid pEY.33K creating plasmid pEY.33Kd3 (Fig. 2A)*. iv)* *pEY.33Kd4*. A 360 bp fragment was amplified from by PCR using the primers P1 and P9 (Table 1), and plasmid EY.33K DNA as a template. Similarly, a 375 bp fragment was amplified by PCR using the primers P4and P10 (Table 1), and plasmid pEY.33K DNA as a template. In a third PCR reaction, two PCR fragments that have 9 bp of internal overlap were annealed and external primers P1 and P4 (Table 1) were used to PCR across to give a 765 bp amplicon. This PCR product was digested with *Eco*RI-*Bam*HI and ligated to *Eco*RI-*Bam*HI digested plasmid pEYFP-N1 creating plasmid pEY.33Kd4 (Fig. 2A). *v)* *pEY.33Kd5.* A 485 bp fragment was amplified by PCR using the primers P1 and P11 (Table 1), and plasmid pEY.33K DNA as a template. Similarly, a 905 bp fragment was amplified by PCR using the primers P12 and P13 (Table 1), and plasmid pEY.33K DNA as a template. In a third PCR reaction, two PCR fragments that have 9 bp of internal overlap were annealed and external primers P1 and P4 (Table 1) were used to PCR across to give a 765 bp amplicon. This PCR product was digested with *Eco*RI-*Bam*HI and ligated to *Eco*RI-*Bam*HI digested plasmid pEYFP-N1 creating plasmid pEY.33Kd5 (Fig. 2A). *vi)* *pEY.33Kd6*. A 610 bp fragment was amplified by PCR using the primers P1 and P14 (Table 1), and plasmid EY.33K DNA as a template. Similarly, a 660 bp fragment was amplified by PCR using the primers P15 and P13 (Table 1), and plasmid pEY.33K DNA as a template. In a third PCR reaction, two PCR fragments that have 9 bp of internal overlap were annealed and external primers P1 and P4 (Table 1) were used to PCR across to give a 765 bp amplicon. This PCR product was digested with *Eco*RI-*Bam*HI and ligated to *Eco*RI-*Bam*HI digested plasmid pEYFP-N1 creating plasmid pEY.33Kd6 (Fig. 2A). *vii) pEY.33Kd7.* A 730 bp fragment was amplified by PCR using the primers P1 and P16 (Table 3.1), and plasmid EY.33K DNA as a template. Similarly, a 525 bp fragment was amplified from by PCR using the primers P17 and P13 (Table 1), and plasmid EY.33K DNA as a template. In a third PCR reaction, two PCR fragments that have 9 bp of internal overlap were annealed and external primers P1 and P18 (Table 1) were used to PCR across to give a 765 bp amplicon. This PCR product was digested with *Eco*RI-*Bam*HI and ligated to *Eco*RI-*Bam*HI digested plasmid pEYFP-N1 creating plasmid pEY.33Kd7 (Fig. 2A).

***d) Plasmids pEY.33Kd6a, pEY.33Kd6b, pEY.33Kd6c****.* i) A 597 bp *Sac*II-*Blp*I fragment of plasmid pC.33Kd6a to 4.9 kb *Sac*II-*Blp*I fragment of pEY.33K plasmid creating plasmid pEY.33Kd6a (200-211 amino acid deletion). ii) A 764 bp fragment was amplified from by PCR using the primers P1 and P4 (Table 1), and plasmid pC.33Kd6b DNA as a template. This PCR product was digested with *Eco*RI-*Bam*HI and ligated to *Eco*RI-*Bam*HI digested EYFP-N1 plasmid creating the plasmid pEY.33Kd6b (Fig. 2A). iii) A 686 bp fragment was amplified from by PCR using the primers P1 and P18 (Table 1), and plasmid pEY.33K DNA as a template. Similarly, a 660 bp fragment was amplified from by PCR using the primers P13 and P19 (Table 1), and plasmid pEY.33K DNA as a template. In a third PCR reaction, both amplified fragments were annealed and external primers P1 and P13 (Table 1) were used to PCR across to give a 1.3 kb amplicon. This PCR product was digested with *Eco*RI-*Bam*HI and ligated to *Eco*RI-*Bam*HI digested EYFP-N1 plasmid creating the plasmid pEY.33Kd6c (Fig. 2A)

***e) Plasmid pC.33Kd6, pC.33Kd6a, pC.33Kd6b and pC.33Kd6c****.* i) A 683 bp *Sac*II-*Eco*RI fragment of plasmid pEY.33Kd6 was ligated to 5.4 Kb *Sac*II-*Eco*RI fragment of plasmid pC.33K creating plasmid pC.33Kd6 (Fig. 2A). ii) A 600 bp fragment was amplified from by PCR using the primers P20 and P21 (Table 1), and plasmid pC.33K DNA as a template. Similarly, a 373 bp fragment was amplified by PCR using the primers P22 and P23 (Table 1), and plasmid pC.33K DNA as a template. In a third PCR reaction, both amplified fragments were annealed and external primers P20 and P24 (Table 1), were used to PCR across to give a 804 bp amplicon. This PCR product was digested with *Sac*II-*Eco*RI and ligated to 5.4 kb *Sac*II-*Eco*RI digested pC.33K creating plasmid pC.33Kd6a (Fig. 2A). iii) A 600 bp fragment was amplified from by PCR using the primers P20 and P25 (Table 1), and plasmid pC.33K DNA as a template. Similarly, a 300 bp fragment was amplified from by PCR using the primers P22 and P26 (Table 1), and plasmid pC.33K DNA as a template. In a third PCR reaction, both amplified fragments were annealed and external primers P20 and P24 (Table 1), were used to PCR across to give a 753 bp amplicon. This PCR product was digested with *Sac*II-*Eco*RI and ligated to *Sac*II-*Eco*RI digested pC.33K creating plasmid pC.33Kd6b (Fig. 2A). iv) A 817 bp fragment was amplified from by PCR using the primers P20 and P24 (Table 1), and plasmid pEY.33K (230-240d) DNA as a template. This PCR product was digested with *Sac*II*-Eco*RI and ligated to 5.4 kb *Sac*II*-Eco*RI digested pC.33K creating plasmid pC33Kd6c (Fig. 2A).

***f) Plasmid pNLS.GFP/βgal*.** A 60 bp fragment amplified by PCR using primers (P57, P58) and plasmid pC.33K DNA, digested with *Sac*II-*Nhe*I and ligated to *Sac*II-*Nhe*I digested plasmid pGFP/βgal creating plasmid pNLS.GFP/βgal (Fig. 2A).

***g) Plasmid pC.33Ksr.*** A 238 bp fragment was amplified by PCR using primers (P51,P56) and plasmid pC.33K DNA. Similarly, a 259 bp fragment was amplified by PCR using primers (P52,P55) and plasmid pcDNA splice 33K as template )plasmid DNA. The PCR products were run on Agarose gel and the DNAs were gel purified using gel extraction kit (Fermentas). In a third PCR reaction, both amplified fragments were annealed and external primers P55 and P56 (Table 1) were used to PCR across to give a 473 bp amplicon. This 437 bp PCR product was gel purified. Next, a 280 bp fragment was PCR amplified using primers (P53, P56) and 473 bp fragment as a DNA template. Similarly, a 219 bp fragment was PCR amplified using primers (P54 and P55) and 473 bp fragment as a DNA template. Both amplified fragments were annealed and external primers P55 and P56 (Table 1) were used to PCR across to give a 473 bp amplicon. This 473 bp PCR product was gel purified, digested with *Asc*I and *Kpn*I and ligated to *Asc*I and *Kpn*I digested pC. 33K plasmid DNA creating plasmid pC.33Ksr (Fig. 3).

**CONSERVED ARGININES OF RS MOTIF OF BOVINE ADENOVIRUS-3 33K PROTEIN ARE IMPORTANT FOR TRANSPORTIN -3 MEDIATED NUCLEAR TRANSPORT AND VIRUS REPLICATION**

**Table 1** **Sequence of primers for the construction of 33K deletion plasmids**

Primer Sequence

P1 5’-GGAATTCTGATGAAACCCCGCAGCATGTCG-3’

P2 5’-GGAATTCTGATGTCGGCAGCCGGGCCT-3’

P3 5’-GGAATTCTGATGATTTCGATACCCCGCGA-3’

P4 5’-CGGGATCCCGGGCGGGTCCGGATTCGTC-3’

P5 5’-GGATTTCCAAGTCTCCAC-3’

P6 5’-CATCATATGCAGGTCCTCCTC-3’

P7 5’-GCTTCTCTGAATCCCACCGC-3’

P8 5’-GGAATTCTGAATGGCTTCTCTGAATCCCAC-3’

P9 5’-AGGGCGAGGCTTGCCCTGGCCCTTCTTG-3’

P10 5’-CAGGGCAAGCCTCGCCCTCCTCCTCTT-3’

P11 5’-GAGAGTGGGCGCGCTGGCACTCCGC-3’

P12 5’-GCCAGCGCGCCCACTCTCTATGCCATAT-3’

P13 5’-GTCCTCGATGTTGTGGCGG-3’

P14 5’-GGTTCGCTGAAAGATCAGCTCCCGAAGCTT-3’

P15 5’-CTGATCTTTCAGCGAACCCTAGCAGACT-3’

P16 5’-GGCGGGTCCGAGCTGTTCCTCCCTTGTTGT-3’

P17 5’-GAACAGCTCGGACCCGCCTAAGAATTCC-3’

P18 5’-GGTTCGCTGGCGCGTCA G TGAACGTA AG-3’

P19 5’-CTGACGCGCCAGCGAACCCTA GCAGAC T-3’

P20 5’-GGAATTCCATATGATGAAACCCCGCAGCATGTCG-3’

P21 5’-CCGCTGAGCGATCA GCTCCCGAAGCTTG-3’

P22 5’-GAAGGCACAGTCGAGGCT-3’

P23 5’-GAGCTGATCGCTCAGCGGTGTCACCTC-3’

P24 5’-GGAATTCCTTAGGCGGGTCCGGATTCG-3’

P25 5’-GTGGTAGAGGAGAG TGGGAAAGATCAGCT-3’

P26 5’-CCCACTCTCCTCTACCACAACAAGGAGGAA-3’

P51 5’- AAATGGATCCTTAGGTTCACTGACGGGC-3

P52 5’- GCAGCTGCCCGTCAGTGAACCTAAGGATCC-3

P53 5’- ATTCCAACAAAGTGGCGCTCAGCGG-3

P54 5’-CGCCACTCAGAGCAAAAAGAGC-3

P55 5’-TTGAGGTGACACCGCTGAGCGCCACTTTG-3

P56 5’-ATTCCAGCACACTGGCGGCCGTTA-3

P57 5’-TCCCCGCGGATGCCCACTCTCTATGCCATATTCC-3

P58 5’-CTAGCTAGCCATTTCTTGCGCGCCCGCGGGGAGC-3’

________________________________________________________________
